# Supplementary material for: Endoscopic Ultrasound-Guided Pancreatic Tissue Sampling: Lesion Assessment, Needles, and Techniques
Source: Medicina (Kaunas). 2024 Dec 7;60(12):2021. doi: 10.3390/medicina60122021 (PMC11727853; doi:10.3390/medicina60122021)
Supplement: Supplementary file 1 [file medicina-60-02021-s001.zip › supplementary document S1_final.pdf]

Supplementary document s1

Searching on Pubmed	
**Search number**	**Query**
#1	EUS-guided OR fine-needle biopsy (FNB) AND random* Sort by: Most Recent " "EUS-guided"[MeSH Terms] OR ("EUS"[All Fields] AND "guided"[All Fields]) OR "EUS guided"[All Fields] EUS-guided: "eus guided"[MeSH Terms] OR ("eus"[All Fields] AND "guided"[All Fields]) OR "eus guided"[All Fields]
#2	("biopsy, fine needle"[MeSH Terms] OR ("biopsy"[All Fields] AND "fine needle"[All Fields]) OR "fine-needle biopsy"[All Fields] OR ("fine"[All Fields] AND "needle"[All Fields] AND "biopsy"[All Fields]) OR "fine needle biopsy"[All Fields]) AND "FNB"[All Fields]

Searching on Pubmed	
**Search number**	**Query**
#1	pancreatic cyst [MeSH Terms]
#2	"cyst pancreas"[Title/Abstract] OR "cystic pancreatic lesions "[Title/Abstract] OR "intraductal papillary mucinous neoplasm "[Title/Abstract] OR "mucinous cystic neoplasm"[Title/Abstract] OR "pancreatic cystic lesions"[Title/Abstract] OR "pancreatic cystic tumor"[Title/Abstract] OR "Pancreatic Intraductal Neoplasms"[Title/Abstract]
#3	"endoscopic ultrasound"[Title/Abstract] OR "diagnostic imaging"[Title/Abstract] OR "endoscopic ultrasound guided fine needle aspiration"[Title/Abstract] OR "EUS FNA"[Title/Abstract] OR "cyst fluid analysis"[Title/Abstract] OR "chemical analysis"[Title/Abstract] OR "tumor biomarker"[Title/Abstract] OR "tumor marker"[Title/Abstract] OR "carcinoembryonic antigen"[Title/Abstract] OR "CEA"[Title/Abstract] OR "glucose"[Title/Abstract] OR "intracystic glucose"[Title/Abstract] OR "pancreatic cyst fluid glucose"[Title/Abstract] OR "cytology"[Title/Abstract] OR "cytopathologic assessment"[Title/Abstract] OR "molecular marker"[Title/Abstract] OR "next generation sequencing"[Title/Abstract] OR "KRAS"[Title/Abstract] OR "GNAS"[Title/Abstract] OR "DNA mutation "[Title/Abstract] OR "Endoscopic ultrasound guided through the needle micro forceps"[Title/Abstract] OR "EUS TTNB"[Title/Abstract] OR "microforceps"[Title/Abstract] OR "microforceps biopsy"[Title/Abstract] OR "EUS MFB"[Title/Abstract] OR "forceps biopsy"[Title/Abstract] OR "fine needle biopsy"[Title/Abstract] OR "EUS FNB"[Title/Abstract] OR "micro forcep"[Title/Abstract] OR "moray micro forceps"[Title/Abstract] OR "through the needle"[Title/Abstract] OR "endomicroscopy"[Title/Abstract] OR "EUS guided needle based confocal laser endomicroscopy"[Title/Abstract] OR "EUS nCLE"[Title/Abstract] OR "contrast enhanced EUS"[Title/Abstract] OR "contrast enhanced harmonic EUS"[Title/Abstract] OR "CE EUS"[Title/Abstract] OR "CH EUS"[Title/Abstract] OR "CEH EUS"[Title/Abstract]
#4	(#1 OR #2) AND #3

Searching on Embase	
**No.**	**Query**

#1	'pancreatic cyst':ti,ab,kw OR 'cyst pancreas':ti,ab,kw OR 'cystic pancreatic lesions':ti,ab,kw OR 'intraductal papillary mucinous neoplasm':ti,ab,kw OR 'mucinous cystic neoplasm':ti,ab,kw OR 'pancreatic cystic lesions':ti,ab,kw OR 'pancreatic cystic tumor':ti,ab,kw OR 'pancreatic intraductal neoplasms':ti,ab,kw
#2	'pancreas cyst'/exp
#3	'endoscopic ultrasound':ti,ab,kw OR 'diagnostic imaging':ti,ab,kw OR 'endoscopic ultrasound guided fine needle aspiration':ti,ab,kw OR 'eus fna':ti,ab,kw OR 'cyst fluid analysis':ti,ab,kw OR 'chemical analysis':ti,ab,kw OR 'tumor biomarker':ti,ab,kw OR 'tumor marker':ti,ab,kw OR 'carcinoembryonic antigen':ti,ab,kw OR 'cea':ti,ab,kw OR 'glucose':ti,ab,kw OR 'intracystic glucose':ti,ab,kw OR 'pancreatic cyst fluid glucose':ti,ab,kw OR 'amylase':ti,ab,kw OR 'cytology':ti,ab,kw OR 'cytopathologic assessment':ti,ab,kw OR 'molecular marker':ti,ab,kw OR 'next generation sequencing':ti,ab,kw OR 'kras':ti,ab,kw OR 'gnas':ti,ab,kw OR 'dna mutation':ti,ab,kw OR 'endoscopic ultrasound guided through the needle microforceps':ti,ab,kw OR 'eus ttnb':ti,ab,kw OR 'microforceps':ti,ab,kw OR 'microforceps biopsy':ti,ab,kw OR 'eus mfb':ti,ab,kw OR 'forceps biopsy':ti,ab,kw OR 'fine needle biopsy':ti,ab,kw OR 'eus fnb':ti,ab,kw OR 'micro forcep':ti,ab,kw OR 'moray micro forceps':ti,ab,kw OR 'through the needle':ti,ab,kw OR 'endomicroscopy':ti,ab,kw OR 'eus guided needle based confocal laser endomicroscopy':ti,ab,kw OR 'eus ncle':ti,ab,kw OR 'contrast enhanced eus':ti,ab,kw OR 'contrast enhanced harmonic eus':ti,ab,kw OR 'ce eus':ti,ab,kw OR 'ch eus':ti,ab,kw OR 'ceh eus':ti,ab,kw
#4	(#1 OR #2) AND #3
#5	#4 AND 'Article'/it
