# Supplementary material for: Endoscopic Ultrasound-Guided Pancreatic Tissue Sampling: Lesion Assessment, Needles, and Techniques
Source: Medicina (Kaunas). 2024 Dec 7;60(12):2021. doi: 10.3390/medicina60122021 (PMC11727853; doi:10.3390/medicina60122021)
Supplement: Supplementary file 1 [file medicina-60-02021-s001.zip › supplementary document S2 (Template of EUS reporting_final).pdf]

Template of EUS reporting for pancreatic lesions:

A. Pre-procedural details of the patient

1. Name
2. Age
3. Sex
4. ID number
5. Date of procedure
6. Outpatient/inpatient examination
7. Date of admission (if applicable)
8. Date of discharge (if applicable)
9. CCI index (Charlson comorbidity index)
10. Presence of comorbidities
11. Number of comorbidities (if present)
12. ASA class (American Society of Anesthesiologists)
13. Past medical history
14. Anti-platelet/anti-coagulant use
15. Past surgical history
16. Sedation given
    - What type of sedation (GA/conscious)
    - Dose
17. Pre-procedural preparation
    - Duration of NPO
    - Bowel preparation
18. Antibiotics given
    - When has it been given (pre/peri/post procedure)
    - Dose and duration
19. Documentation of informed consent

B. Endoscopist details:

1. Name of primary endoscopist
2. Name(s) of assistant/trainee
3. Name(s) of nurse

C. Relevant investigations:

1. Complete hemogram
2. Liver function tests
3. PT-INR
4. Renal function tests
5. CA 19-9 (if applicable)
6. CEA (if applicable)
7. Relevant CT findings
8. Relevant MRI/MRCP findings

D. **Indication of Endoscopic ultrasound** (mention complete details)

I. Diagnostic indication

II. Therapeutic indication

E. Peri-procedural EUS details:

1. Type of EUS scope (linear/radial)
2. Make of EUS scope
3. Adequacy and extent of examination
4. Description

- Relevant landmarks Lesion(s)
- Size
- Sonographic characteristics
- Pertinent positives/negatives Tumour staging (TNM)

Note: distant metastasis cannot be ruled out

F. Relevant findings while EUS assessment of pancreatic lesions:

1. Solid/cystic lesions of pancreas

i. Number of lesions
ii. Size of lesions
iii. Location (head/ neck/ uncinate/ body/ tail)
iv. Morphology
v. Biliary dilatation (mention size[mm])
vi. PD (diameter mm)
vii. Relevant vasculature (relation with SMA/SMV/PV/CA/others)
viii. Associated lymph nodes (size, contours, echogenicity);
ix. Mention if EUS-FNA/FNB done of the LN:

x. If solid lesion:

- Depth of tumour (T and N staging)
- Ascites
- Pleural effusion
- Distance of MPD from the lesion (mm)
- Upstream MPD dilatation
- Upstream atrophy noted
- Contrast enhanced EUS findings
- EUS-elastography findings ; mention strain ratio
- IMPRESSION:

xi. If Cystic lesions:

- Number
- Location
- Septations
- Mural nodules
- Diameter (2 axis)
- Compartment (if present) (size of largest compartment)

- Enhancing cyst wall
- MPD communication
- IMPRESSION :

2. **Pancreatic parenchyma**

   i. Parenchyma:

   - Echogenicity
   - Homogenous/heterogenous
   - Atrophy
   - Stranding
   - Lobularity
   - Cysts
   - Calcifications
   - Hyperechoic foci
   - Hyperechoic strands
   - Fatty pancreas

   ii. Pancreatic duct

   - Anatomy
   - Contour
   - Diameter (mm) (2 axis)
   - Echogenicity of the duct
   - Stricture
   - Stent noted
   - Filling defect noted
   - Pancreas divisum noted

3. **Biliary anatomy**

   i. CBD (diameter mm)
   ii. CHD (diameter mm)
   iii. Confluence (primary) [patent/non patent]
   iv. IHBRD (present or absent)
   v. Cystic duct
   vi. Gall bladder (wall thickness, stones, oedema; size; location)
   vii. Relevant vascular anatomy
   viii. If any stent noted
   ix. Stones and/or stricture in CBD

4. **Ampulla description**

5. **Sampling method and sample quality**

   - Needle type and gauge

- Make of needle
- Number of needle passes
- Number of actuations
- Technique (fanning/torque/door knocking)
- Suction used (wet/modified wet/dry)
- Stylet used (slow pull)
- Onsite evaluation used (ROSE/MOSE/VOSE/SOSE)
- FNA/FNB performed using contrast enhanced or elastography guided
- Tissue expressed using:

6. **Cyst fluid analysis:**

    - String test (if done)
    - Glucose
    - CEA levels
    - Additional markers

7. **EUS-TTNB used in cyst analysis** (specify all details of the procedure)

    - timing of cyst aspiration
    - forceps preloading
    - number of passes and bite per pass
    - specimen handling

8. **Intraprocedural adverse events (specify all details)**

9. **Biliary drainage performed in same / separate session (mention details)**

10. **Additional procedures performed for tissue sampling (mention details)**

G. Post procedural details:

1. Final diagnosis and relevant finding
2. Management of complications (if applicable)
3. Follow-up schedule
4. Recommendations regarding the resumption of anti-platelet /anti-coagulant
5. Final histopathological diagnosis
