# Supplementary material for: Endoscopic Ultrasound-Guided Pancreatic Tissue Sampling: Lesion Assessment, Needles, and Techniques
Source: Medicina (Kaunas). 2024 Dec 7;60(12):2021. doi: 10.3390/medicina60122021 (PMC11727853; doi:10.3390/medicina60122021)
Supplement: Supplementary file 1 [file medicina-60-02021-s001.zip › Supplementary table_final.pdf]

Supplementary Table S1: Steps to perform advanced EUS imaging (Contrast enhanced harmonic endoscopic ultrasound) during evaluation of a suspected pancreatic lesion

Contrast enhanced harmonic endoscopic ultrasound:

The technique is based on use of a second-generation blood-pool ultrasound contrast agent [UCA] injected through a peripheral vein, which subsequently can be visualized through the microcirculation in the early arterial and late venous phase, especially when used with contrast harmonic imaging techniques (low mechanical index [MI] techniques). Contrast agents can be used as a Doppler signal enhancer (high mechanical index techniques) as well as used for time intensity curve (TIC) analysis. UCA agents are very safe with low incidence of side effects, as a result, it is not necessary to perform laboratory tests (LFT, KFT) prior to their administration.

1. Pre-contrast examination should first identify the best position of the patient, identification of the target lesion and optimal scan plane along the axis of the respiratory movements (usually longitudinal).
2. Quiet breathing and breath suspension in neutral are preferred.
3. Both B-mode and contrast image are put side by side on the EUS machine screen with the pancreatic lesion in focus.
4. Insert cannula usually on the left arm, preferably the antecubital vein, to avoid interaction of the injector with the right sided examiner. Ideally, venous access should be made with a 20 G or larger to avoid microbubble destruction during passage through the cannula, with its length as short as possible.
5. A three way stop cock may be valuable if multiple injections are anticipated (facilitating sequential contrast and saline flush administration, without removal of either syringe).
6. The **three contrast agents** (second generation ultrasound contrast agents) commonly available for use are **SonoVue** (Bracco diagnostics; Sulfurhexaflouride; 2-3 um; use with saline), **Sonazoid** (GE healthcare; perflourobutane, 1-2 um, use with water) and **Definity** (Bristol-myers Squibb medical imaging; octoflouropropane; 1.1-3.3 um, used with vialmix agitation). The former 2 are more commonly used in day-to-day clinical practice. These contrast agents resonate under low acoustic power and generate a second harmonic component, providing at least several minutes of contrast effect. Sonazoid, commonly used in Japan, facilitates early, late and Kupffer phase assessment after 10-30 s, 30-120 s, and 10 min of contrast injection. In short, all UCA are blood pool agents.
7. After careful stabilization of the image, the system software is switched to a low MI contrast specific mode and SonoVue is then injected (2.4–4.8 ml) through a peripheral vein. Injection bolus is usually 1-2 ml/second. High pressure is avoided to prevent microbubble destruction. After contrast is injected, 10 ml saline bolus is given as 2 ml/second to flush the cannula.
8. Two phases exist for pancreatic imaging: an early (arterial) phase (starting from approximately 10 s to 30 s) and a venous (late) phase (starting from approximately 30 s to 120 s). After the bolus injection of a microbubble UCA, time-intensity curves (TICs) analysis is used as a quantitative analysis of perfusion imaging, during the early (wash-in) and late (wash-out).
9. Usually contrast agent is suspected in 2 ml water and intravenously injected at 0.015 ml/kg; and post administration, pancreatic lesion is assessed for 90-120 second and image movie data is recorded.
10. Post procedure, patient is usually monitored for 2 hours and cannula can be safely removed thereafter.

Supplementary Table S2: Steps to perform advanced EUS imaging (Endoscopic ultrasound guided elastography) during evaluation of a suspected pancreatic lesion

Endoscopic ultrasound guided elastography:

EUS-E measures the tissue displacement as a key step to measure the elasticity of the tissue. Tissue elastography is obtained when tissue compression is applied in the region of interest (ROI). Then, the deformity or "strain" is measured using a transparent color overlay on the B-mode image.

1. Basic position and pre-procedural instruction remain the same as described in supplementary table 1.
2. Keep the image (pancreatic lesion) in focus on the screen with both B-mode image and elastography image.
3. The best output is obtained when the lesion of interest covers 25–50% of the ROI.
4. **Qualitative EUS-E:** Elastography pattern is depicted (scale of 1-255) by superimposing a transparent color pattern on conventional B-mode imaging. Hard tissue is depicted in dark blue, intermediate in green, medium in yellow, and soft tissue in red.
5. **Quantitative EUS-E:** This entails strain ratio (SR) and strain histogram (SH).
    - **Strain ratio (SR)** is an extension of the primary qualitative elastography data. Area A is delineated by including as much of the target lesion as possible. A soft area (red) outside the lesion is marked as area B, usually the gut wall. The ratio is calculated as B/A. Although the color helps guide the choice of the area, the actual ratio is calculated from raw strain data. **Usually, a strain ratio > 10 is considered malignant.**
    - **Strain histogram (SH)** requires drawing the largest box that can fit within the lesion boundary of ROI. The X-axis then represents the tissue's stiffness (from 0 being hardest to 255 being softest). Next, the data of the strain patterns are used to generate a histogram using Gaussian distribution. It gives additional information about the mean strain, percentage of hard tissue (blue zone), and the relative comparison of the blue patches to the surrounding. **An SH < 50 is considered to be malignant.**
6. **A few tips and tricks are to be kept in mind while performing EUS-E:**
    - the size of the ROI should be adequately large to include both the pathological lesion and the surrounding normal tissue for reference;
    - large blood vessels should be avoided as it can lead to a false impression of softness due to large displacement
    - the curved array transducer can lead to differential compression at the center and the lateral part, and thus, narrowing the ROI sector can help improve uniformity
    - in the freeze mode, the strain graph can be used to help select the best frame for interpretation
    - ROI can be resized even on a frozen image frame
    - lesions with more vessels, necrosis, or calcification should be interpreted with caution while using EUS-E because of non-uniform stress distribution
7. **Sheer wave elastography**: While strain elastography uses compression to deform tissues to measure "strain", SWE uses acoustic radiation force (ARF) to initiate shear wave propagation and measure propagation speed to gauge the elasticity of the tissue. A "push" pulse is applied to the tissue using ARF and the tissue displacement is measured in the direction perpendicular to the shear wave propagation. The speed of the shear wave propagation, vs, is related to the stiffness of the tissue. The elastic modulus, E, of the tissue is proportional to the square of the shear wave propagation speed.

Supplementary Table S3: Studies reporting complementary roles of use of contrast enhanced harmonic EUS for assessment and management of pancreatic lesions

author (year)	diagnosis	number	aim of study	results
**Assessing responses to chemotherapy**				
Yamashita et al (2023) (A)	PC	94	to assess pathological response after NAC in pancreatic cancer patients	• Divided responses of CH-EUS into early vs late phase (enhancement pattern) and rich and poor tumor vascularity.<br>• Multivariate analysis: rich tumor vascularity strongest factor to determine chemosensitivity<br>• Resectable cases: enhancement pattern determined chemosensitivity
Dahel et al (2023) (B)	advanced PDAC	16	assess biological effect of nab-paclitaxel and gemcitabine using vascularization patterns	No difference in vascularization or elasticity of PC after chemotherapy
Emori et al (2022) (C)	unresectable PC		predicting efficacy of first line CT (gem/nab paclitaxel)	• Characterized in 4 groups based on vascularity in early and late phases of contrast enhancement<br>• Group D (rich vascularity in both phases): longest overall and progression free survival ($p<0.001$)<br>• Correlation existed between CH-EUS and CECT scan
Tanaka et al (2019) (D)	PC	23	evaluate response to CT	• Super responders: >50% reduction in ca 19-9 levels after CT<br>• 9 cases were super responders<br>• Detection of avascular areas by CH-EUS after CT predicts long term survival
**Guidance and monitoring during EUS-RFA**				
Choi et al (2020) (E)	pancreatic tumours (NF-NET 13; insulinoma 1; SPN 2; left adrenal adenoma 1; left adrenal metastasis 1)	19	determine extent of ablation using EUS-RFA and guide further management in residual disease	• Technical success: 100% (19/19) using CH-EUS guidance<br>• After first RFA session, 7 had complete disappearance of tumor enhancement<br>• 12 cases: additional second session needed (showing incompete response)<br>• 1 adverse event only (5.7%)

Abbreviations: EUS endoscopic ultrasound; CH-EUS contrast enhanced harmonic EUS; PC pancreatic cancer; PDAC pancreatic ductal adenocarcinoma; CT chemotherapy; CECT contrast enhanced computed tomography; NAC neo-adjuvant chemotherapy; NF-NET non-functional neuroendocrine tumors; SPN solid-pseudopapillary neoplasm; RFA radiofrequency ablation

References:

A. Yamashita Y, Ashida R, Kojima F, Okada KI, Kawai M, Yamazaki H, Tamura T, Kawaji Y, Tamura T, Hatamaru K, Itonaga M, Kitano M. Utility of contrast-enhanced harmonic endoscopic ultrasonography for prediction of pathological response after neoadjuvant chemotherapy in patients with pancreatic cancer. Pancreatology. 2023 Dec;23(8):1014-1019.

B. Dahel Y, Chanez B, Zemmour C, Piana G, Mitry E, Giovannini M. Assessment of biological effect of nab-paclitaxel combined with gemcitabine, using contrast enhanced ultrasonography and elastography, in advanced pancreatic ductal carcinoma: A single-center pilot study. Endosc Ultrasound. 2023 Mar-Apr;12(2):273-276.

C. Emori T, Ashida R, Tamura T, Kawaji Y, Hatamaru K, Itonaga M, Yamashita Y, Shimokawa T, Higashino N, Ikoma A, Sonomura T, Kawai M, Kitano M. Contrast-enhanced harmonic endoscopic ultrasonography for predicting the efficacy of first-line gemcitabine and nab-paclitaxel chemotherapy in pancreatic cancer. Pancreatology. 2022 May;22(4):525-533.

D. Tanaka H, Kamata K, Takenaka M, Yoshikawa T, Ishikawa R, Okamoto A, Yamazaki T, Nakai A, Omoto S, Minaga K, Yamao K, Sakurai T, Watanabe T, Nishida N, Chiba Y, Kitano M, Kudo M. Contrast-enhanced harmonic endoscopic ultrasonography for evaluating the response to chemotherapy in pancreatic cancer. Dig Liver Dis. 2019 Aug;51(8):1130-1134.

E. Choi JH, Seo DW, Song TJ, Park DH, Lee SS, Lee SK, Kim MH. Utility of Contrast-Enhanced Harmonic Endoscopic Ultrasound for the Guidance and Monitoring of Endoscopic Radiofrequency Ablation. Gut Liver. 2020 Nov 15;14(6):826-832.

Supplementary Table S4: Studies comparing EUS-Elastography guided tissue acquisition versus standard EUS-FNA/B for evaluation of pancreatic masses

Author, year	Study design	Comparison arms	Sensitivity %	Specificity %	Accuracy %	PPV %	NPV %	Adverse events %
Nayak et al (2024) (42)	RCT	EUS-E guided FNA vs standard EUS-FNA (20 vs 20)	87.5% vs 82.3%	100% vs 100%	90% vs 85%	100% vs 100%	62.9% vs 54.59%	3 (15%) vs 2 (10%)
Mayerle et al (2016) (A)	Prospective	EUS-E alone versus EUS-FNA 91 overall (cross-over)	96% vs 94%	34% vs 64%	84% vs 87%	-	-	-
Kongkam (2015) (B)	Prospective	EUS-FNA vs EUS-FNA + strain ratio of EUS-E	90% vs 95.2%	100% vs 71.4%	92.9% vs 89.3%	100% vs 90.9%	80% vs 83.3%	-
Gheorghiu et al (2022) (43)	Prospective	EUS-E guided FNA vs standard EUS-FNA (60 vs 60)	89.5% vs 93%	100% vs 100%	90% vs 93.3%	100% vs 100%	33.3% vs 42.9%	-

Abbreviations: EUS endoscopic ultrasound; FNA fine needle aspiration; FNB fine needle biopsy; EUS-E endoscopic ultrasound guided elastography; RCT randomized controlled trial; PPV positive predictive value; NPV negative predictive value

References:

A. Mayerle J, Beyer G, Simon P, Dickson EJ, Carter RC, Duthie F, Lerch MM, McKay CJ. Prospective cohort study comparing transient EUS guided elastography to EUS-FNA for the diagnosis of solid pancreatic mass lesions. Pancreatology. 2016 Jan-Feb;16(1):110-4.

B. Kongkam P, Lakananurak N, Navicharern P, Chantarojanasiri T, Aye K, Ridtitid W, Kritisin K, Angsuwatcharakon P, Aniwan S, Pittayanon R, Sampatanukul P, Treeprasertsuk S, Kullavanijaya P, Rerknimitr R. Combination of EUS-FNA and elastography (strain ratio) to exclude malignant solid pancreatic lesions: A prospective single-blinded study. J Gastroenterol Hepatol. 2015 Nov;30(11):1683-9.

Supplementary Table S5: Studies (randomized controlled trials and meta-analysis) on the use of suction or stylet techniques for performing EUS-guided tissue acquisition

Author (year)	Design of studies	EUS-TA	Suction technique	Number of patients	Tissue adequacy	Blood contamination	Cellularity	Diagnostic accuracy
**RCTs comparing WEST versus slow pull technique using EUS-FNB (end cutting needles)**								
Ladd et al (2022) (94)	RCT	EUS-FNB (end cutting)	DS vs SP vs WEST	20 vs 18 vs 17	First pass: 55% vs 39% vs 35%	score 1: 95% vs 100% vs 100%	mean 3.55 vs 3.28 vs 2.94	-
Crinò et al (2023) (95)	RCT	EUS-FNB (end cutting)	SP vs WEST	102 vs 108	-	mean 2.44 vs 2.09	mean 2.48 vs 2.63	87.1% vs 91.4%
Samanta et al (2023) (96)[#]	RCT	EUS-FNB (end cutting)	SP vs WEST	85 vs 85	97.6% vs 97.6% (p=1)	mean 2.19 vs 2.24	mean 2.82 vs 2.85	98.8% vs 97.6% (p=0.56)
**Meta-analysis on suction techniques for EUS-TA**								
Wang et al (2021) (A)	11 studies (6 RCTs)	EUS FNA/B	SP vs SS	504	SP better (OR 1.91)	SS higher (OR 1.93)	Both similar (OR 0.99)	SP better (OR 1.6)
Nakai et al (2021) (B)	17 studies (7 RCTs)	EUS FNA/B	SP vs SS	1616	-	SS higher (OR 0.48)	Both similar (OR 1.28)	SP better (OR 1.48)
Ramai et al (2021) (C)	6 studies	EUS FNA	WEST vs SS	418	WEST better (OR 3.18)	similar (OR 1.18)	-	similar (OR 3.68)
Giri et al (2023) (D)	16 RCTs	EUS FNA/B	all techniques	2048	WEST best (SUCRA 85.4) > SP>SS>NS	SS highest	WEST best (SUCRA 77.2) >SP>SS>NS	WEST best (SUCRA 80.2) > NS>SS> SP
Capurso et al (2020) (E)	7 RCTs	EUS FNA/B	SP vs suction	475	both similar (OR 0.98)	Suction higher	-	SP better (not significant p value) (OR 0.82)
Facciorusso et al (2023) (98)	9 RCTs	EUS FNB	all techniques	756	WEST best (SUCRA 0.9) > SS=SP	SS highest (SUCRA 0.12)	WEST best (SUCRA 0.89) > SP >SS> NS	WEST best >SP>SS>NS

Abbreviations: EUS endoscopic ultrasound; TA tissue acquisition; FNA fine needle aspiration; FNB fine needle biopsy; WEST wet suction technique; SP slow pull; SS standard suction; NS no suction; RCT randomized controlled trials; OR odds ratio. # this RCT is a conference abstract

References:

A. Wang J, Xu H, Xu C, Feng Y, Zhang X, Zhao S. Stylet Slow-pull Versus Standard Suction for Endoscopic Ultrasound-guided Fine-needle Aspiration/Biopsy of Pancreatic Solid Masses: A Meta-Analysis. *J Clin Gastroenterol*. **2021**;*55*:103-109.
B. Nakai Y, Hamada T, Hakuta R, Sato T, Ishigaki K, Saito K, Saito T, Takahara N, Mizuno S, Kogure H et al. A Meta-analysis of Slow Pull versus Suction for Endoscopic Ultrasound-Guided Tissue Acquisition. *Gut Liver*. **2021**;*15*:625-633.
C. Ramai D, Singh J, Kani T, Barakat M, Chandan S, Brooks OW, Ofosu A, Khan SR, Dhindsa B, Dhaliwal A et al. Wet- *versus* dry-suction techniques for EUS-FNA of solid lesions: A systematic review and meta-analysis. *Endosc Ultrasound*. **2021**;*10*:319-324.
D. Giri S, Afzalpurkar S, Angadi S, Marikanty A, Sundaram S. Comparison of suction techniques for EUS-guided tissue acquisition: Systematic review and network meta-analysis of randomized controlled trials. *Endosc Int Open*. **2023**;*11*:E703-E711.
E. Capurso G, Archibugi L, Petrone MC, Arcidiacono PG. Slow-pull compared to suction technique for EUS-guided sampling of pancreatic solid lesions: a meta-analysis of randomized controlled trials. *Endosc Int Open*. **2020**;*8*:E636-E643

Supplementary Table S6: Randomized controlled trials comparing various actuation number per needle pass during EUS-FNA/B of pancreatic masses

Author (year)	actuation number	EUS needle used	Number of patients	diagnostic accuracy	blood contamination	adequacy rate
Paik et al (2021) (101)	10,15,20,25	EUS-FNA (22 G)	74 cases (suction); 79 cases (no-suction)	15 > 10 (no suction); rest all similar in all groups	15 >20 (suction group); rest all similar	-
Hu et al (2023) (100)	20 vs 40	EUS FNB (22 G; ProCore)	28 vs 27	80% vs 72.7% (p=0.28)	-	60% vs 56.4% (p=0.815)
Kataoka et al (2023) (102)	15 vs 5	EUS-FNB (22 G franseen)	85 vs 85 (cross-over)	83.5% vs 77.7%	median 1.99 vs 1.71mm^2 (p=0.6)	69% vs 31% (p=0.005)
Takahashi et al (2023) (103)	3 vs 12	EUS-FNB (22 G franseen)	110 vs 110 (cross-over)	92.7% vs 94.6%	-	- (higher core tissue with 3 actuation group 71.8% vs 52.7%; p=0.009)

Abbreviations: EUS endoscopic ultrasound; FNA fine needle aspiration; FNB fine needle biopsy; G gauge

Supplementary Table S7: Studies on EUS-guided tissue acquisition using EUS-FNB needles in autoimmune pancreatitis

Study (year)	Study design	Case number	EUS-FNB needle	accuracy	adequacy	number passes	adverse events	IDC 1 criteria	IDC 2 criteria
Jung et al (2015) (A)	Retrospective	62 (in EUS group)	22 G reverse bevel (20 cases)	70%	-	mean 3.10	3.2% (mild AP)	-	-
Kurita et al (2020) (152)	RCT	50 vs 51	22 G Franssen vs 20 G forward bevel	78.2% vs 41.8%	88% vs 68%	-	2% vs 2%	56% vs 26%	22% vs 20%
Tsutsumi et al (2021) (B)	Retrospective	14	21 G Menghini	-	100%	median 4 (3-5)	14%	36%	29%
Lee et al (2017)# (C)	Retrospective	15 vs 42	19 G FNB vs 22 G reverse bevel	-	100% vs 73.8%	median 2 vs 3	0 vs 0	-	-
Zator et al (2018)# (D)	Retrospective	29	19/22/25 G FNB	79.3%	-	median 2 (1-7)	16%	18/21 (85.7%)	3/21 (14.2%)
Ishikawa et al (2020) (E)	Prospective	56	22 G Franssen	92.7%	100%	-	4%	58.2%	34.5%
Oppong et al (2020) (F)	Retrospective	24	Fork tip (18); reverse bevel (6)	overall, 50% (78% vs 0)	overall, 87% (94% vs 67%)	overall mean 2.42 (2.55 vs 2)	-	overall, 54% (72% vs 0)	overall, 4% (7% vs 0)
Nogochi et al (2020) (G)	Retrospective	32	19,22, 20 G FNB	17/32 (53.1%)	-	-	12.5%	10 cases	7 cases
Thomsen et al (2022) (H)	Retrospective	15	SharkCore FNB	99.2%	86.7%	-	-	11 cases	8 cases
Notohara et al (2020) (I)	Retrospective	85	Franseen 27; End-cutting 58	-	85.88% (73/85)	median 3 (1-14)	-	22 cases	23 cases
Ishikawa et al (2024) (J)	Prospective	20	19 G Franseen	18/20 (90%)	65%		15%	13 cases	3 cases (15%)
Bellocchi et al (2023)# (153)	Prospective	30	22 G Franseen (7 cases); 22 G Fork tip (32 cases)	85% vs 93.7%	100% vs 100%	-	-	25 cases	11 cases

Abbreviations: EUS endoscopic ultrasound; FNB fine needle biopsy; G gauge; AP acute pancreatitis; IDC international consensus diagnostic criteira; RCT randomized controlled trial;
Note: # are conference abstracts

References:

A. Jung JG, Lee JK, Lee KH, Lee KT, Woo YS, Paik WH, Park DH, Lee SS, Seo DW, Lee SK, Kim MH. Comparison of endoscopic retrograde cholangiopancreatography with papillary biopsy and endoscopic ultrasound-guided pancreatic biopsy in the diagnosis of autoimmune pancreatitis. Pancreatology. 2015 May-Jun;15(3):259-64.

B. Tsutsumi K, Ueki T, Noma Y, Omonishi K, Ohno K, Kawahara S, Oda T, Kato H, Okada H. Utility of a 21-gauge Menghini-type biopsy needle with the rolling method for an endoscopic ultrasound-guided histological diagnosis of autoimmune pancreatitis: a retrospective study. BMC Gastroenterol. 2021 Jan 7;21(1):21.

C. Lee SS, Oh D, Cho DH, Song TJ, Park DH, Seo DW, Lee SK, Kim MH. Su1379 Comparison of 19G Versus 22G Reverse Side-Bevel Needles for Endoscopic Ultrasound-Guided Pancreatic Core Biopsy of Autoimmune Pancreatitis. Gastrointestinal Endoscopy. 2017 May 1;85(5):AB357-8.

D. Zator ZA, Zhu H, Cui M, Kumta NA, Nagula S, DiMaio CJ. Mo1343 small-caliber eus-guided core biopsy needles are a safe and effective means to diagnose autoimmune pancreatitis. Gastrointestinal Endoscopy. 2018 Jun 1;87(6):AB454-5.

E. Ishikawa T, Kawashima H, Ohno E, Suhara H, Hayashi D, Hiramatsu T, Matsubara H, Suzuki T, Kuwahara T, Ishikawa E, Shimoyama Y, Kinoshita F, Hirooka Y, Fujishiro M. Usefulness of endoscopic ultrasound-guided fine-needle biopsy for the diagnosis of autoimmune pancreatitis using a 22-gauge Franseen needle: a prospective multicenter study. Endoscopy. 2020 Nov;52(11):978-985.

F. Oppong KW, Maheshwari P, Nayar MK, Darne A, Parkinson D, Leeds JS, Haugk B. Utility of endoscopic ultrasound-guided fine-needle biopsy in the diagnosis of type 1 autoimmune pancreatitis. Endosc Int Open. 2020 Dec;8(12):E1855-E1861.

G. Noguchi K, Nakai Y, Mizuno S, Hirano K, Kanai S, Suzuki Y, Inokuma A, Sato T, Hakuta R, Ishigaki K, Saito K, Saito T, Hamada T, Takahara N, Kogure H, Isayama H, Koike K. Role of Endoscopic Ultrasonography-Guided Fine Needle Aspiration/Biopsy in the Diagnosis of Autoimmune Pancreatitis. Diagnostics (Basel). 2020 Nov 15;10(11):954.

H. Thomsen MM, Larsen MH, Di Caterino T, Hedegaard Jensen G, Mortensen MB, Detlefsen S. Accuracy and clinical outcomes of pancreatic EUS-guided fine-needle biopsy in a consecutive series of 852 specimens. Endosc Ultrasound. 2022 Jul-Aug;11(4):306-318.

I. Notohara K, Kamisawa T, Kanno A, Naitoh I, Iwasaki E, Shimizu K, Kuraishi Y, Motoya M, Kodama Y, Kasashima S, Nishino T, Kubota K, Sakagami J, Ikeura T, Kawa S, Okazaki K. Efficacy and limitations of the histological diagnosis of type 1 autoimmune pancreatitis with endoscopic ultrasound-guided fine needle biopsy with large tissue amounts. Pancreatology. 2020 Jul;20(5):834-843.

J. Ishikawa T, Yamao K, Mizutani Y, Iida T, Uetsuki K, Shimoyama Y, Nakamura M, Furukawa K, Yamamura T, Kawashima H. A prospective study on the histological evaluation of type 1 autoimmune pancreatitis using endoscopic ultrasound-guided fine needle biopsy with a 19-gauge Franseen needle. J Hepatobiliary Pancreat Sci. 2024 Aug;31(8):581-590.

Supplementary Table S8: Meta-analysis published evaluating the role of intra-cystic glucose and CEA levels in the pancreatic cystic diagnosis

Study (year)	Number of studies	Cut-off level (glucose)	Cut-off level (CEA)	Sensitivity diagnosis mucinous cyst	Specificity diagnosis mucinous cyst
Mohan et al (2022) (172)	7	50 mg/dl	-	glucose (90.1% (87.2%-92.5%))	glucose (85.3% (76.8%-91.1%))
McCarty et al (2021) (170)	8	50 mg/dl	192 ng/ml	glucose 91% (88%-94%)<br>CEA 56% (46%-66%)	glucose 86% (81%-90%)<br>CEA 96% (90%-99%)
Faias et al (2021) (171)	31 (CEA); 4 (glucose)	50 mg/dl	192 ng/ml	glucose 90% (85%-94%)<br>CEA 67% (65%-70%)	glucose 82% (72%-89%)<br>CEA 80% (76%-83%)
Pfluger et al (2023) (174)	10 (CEA); 2 (glucose)	50 mg/dl	192 ng/ml	glucose 93% (89%-96%)<br>CEA 58% (45%-71%)	glucose 76% (59%-93%)<br>CEA 87% (82%-92%)
Guzman-Calderon et al (2022) (173)	6	50 mg/dl	-	91% (88%-93%)	85% (81%-87%)
Khan et al (2022) (A)	15	-	192 ng/ml	60.4% (57.7%-62.9%)	88.6% (85.9%-90.9%)

Abbreviations: CEA carcinoembryonic antigen

References:

A. Khan I, Baig M, Bandepalle T, Puli SR. Utility of Cyst Fluid Carcinoembryonic Antigen in Differentiating Mucinous and Non-mucinous Pancreatic Cysts: An Updated Meta-Analysis. Dig Dis Sci. 2022 Sep;67(9):4541-4548.

Supplementary Table S9: Meta-analysis published on the use of EUS-guided TTNB for pancreatic cystic lesions

Study (year)	Number of studies	Cases (overall)	Technical success	Diagnostic performance	Adequacy	Mean number of passes	Adverse events
Gopakumar et al (2024) (A)	11	575	98.65%	sensitivity 76.6%	Failed tissue acquisition rates 17%	mean 3.22	Intracystic bleeding: 4.02%<br>Infection: 0.94%<br>Pancreatitis: 3.04%
Kovacevic et al (2021) (B)	20	1023	94%	sensitivity 80%	74%	-	Intracystic bleeding: 2.4%<br>Infection: 0.4%<br>Pancreatitis: 3.9%<br>Overall: 5%
Rift et al (2021) (C)	10	99	-	sensitivity 86%	-	mean 2-3	-
Westerveld et al (2020) (D)	8	426	98.2%	72.5%	-	-	Intracystic bleeding: 5%<br>Infection: -<br>Pancreatitis: 2.3%<br>Overall: 7%
Tacelli et al (2020) (E)	9	454	98.5%	69.5%	86.5%	-	Overall: 8.6% (1-23%)
McCarty et al (2020) (F)	11	518	97.12%	82.76%	79.6%	mean 2.47	Intracystic bleeding: 3.61%<br>Infection: -<br>Pancreatitis: 3.94%<br>Overall: 8.26%
Facciorusso et al (2020) (G)	11	490	-	78.8%	85.3%	mean 3.12	Intracystic bleeding: 4%<br>Infection: -<br>Pancreatitis: 2%<br>Overall: 6%
Faias et al (2019) (H)	7	1206	93.2%	73%	-	-	-
Balaban et al (2021) (I)	9	463	98.5%	88.2%	68.6%	-	Intracystic bleeding: 5.61%<br>Infection: 0.64%<br>Pancreatitis: 2.37%<br>Overall: 9.7%
Guzmán-Calderón et al (2020) (J)	8	423	95.6%	74.6%	82.2%	-	Intracystic bleeding: 5.3%<br>Infection: 0.26%<br>Pancreatitis: 2.66%<br>Overall: 10.1%

Abbreviations: EUS endoscopic ultrasound; TTNB through the needle biopsy; NR not reported

References:

A. Gopakumar H, Puli SR. Value of Endoscopic Ultrasound-Guided Through-the-Needle Biopsy in Pancreatic Cystic Lesions. A Systematic Review and Meta-Analysis. *J Gastrointest Cancer*. **2024**;*55*:15-25.

B. Kovacevic B, Antonelli G, Klausen P, Hassan C, Larghi A, Vilmann P, Karstensen JG. EUS-guided biopsy *versus* confocal laser endomicroscopy in patients with pancreatic cystic lesions: A systematic review and meta-analysis. *Endosc Ultrasound.* **2021**;*10*:270-279.

C. Rift CV, Scheie D, Toxværd A, Kovacevic B, Klausen P, Vilmann P, Hansen CP, Lund EL, Hasselby JP. Diagnostic accuracy of EUS-guided through-the-needle-biopsies and simultaneously obtained fine needle aspiration for cytology from pancreatic cysts: A systematic review and meta-analysis. *Pathol Res Pract*. **2021**;*220*:153368.

D. Westerveld DR, Ponniah SA, Draganov PV, Yang D. Diagnostic yield of EUS-guided through-the-needle microforceps biopsy versus EUS-FNA of pancreatic cystic lesions: a systematic review and meta-analysis. *Endosc Int Open.* **2020**;*8*:E656-E667.

E. Tacelli M, Celsa C, Magro B, Barchiesi M, Barresi L, Capurso G, Arcidiacono PG, Cammà C, Crinò SF. Diagnostic performance of endoscopic ultrasound through-the-needle microforceps biopsy of pancreatic cystic lesions: Systematic review with meta-analysis. *Dig Endosc*. **2020**;*32*:1018-1030.

F. McCarty T, Rustagi T. Endoscopic ultrasound-guided through-the-needle microforceps biopsy improves diagnostic yield for pancreatic cystic lesions: a systematic review and meta-analysis. *Endosc Int Open*. **2020**;*8*:E1280-E1290.

G. Facciorusso A, Del Prete V, Antonino M, Buccino VR, Wani S. Diagnostic yield of EUS-guided through-the-needle biopsy in pancreatic cysts: a meta-analysis. *Gastrointest Endosc*. **2020**;*92*:1-8.e3.

H. Faias S, Pereira L, Luís Â, Chaves P, Cravo M. Genetic testing *vs* microforceps biopsy in pancreatic cysts: Systematic review and meta-analysis. *World J Gastroenterol.* **2019**;*25*:3450-3467.

I. Balaban VD, Cazacu IM, Pinte L, Jinga M, Bhutani MS, Saftoiu A. EUS-through-the-needle microbiopsy forceps in pancreatic cystic lesions: A systematic review. *Endosc Ultrasound.* **2021**;*10*:19-24.

J. Guzmán-Calderón E, Martinez-Moreno B, Casellas JA, de Madaria E, Aparicio JR. Endoscopic ultrasound-guided, through-the-needle forceps biopsy for diagnosis of pancreatic cystic lesions: a systematic review. *Endosc Int Open*. **2020**;*8*:E1123-E1133.
